# System of Diseases of the Eye

**Published:** 1898-06

**Authors:** 


**System of Diseases of the Eye.** Edited by WILLIAM F. NORRIS, M.D., and CHARLES A. OLIVER, M.D. Vol. II.—*Examination of the Eye, School Hygiene, Statistics of Blindness, and Antisepsis.* Pp. 556. London and Philadelphia: J. B. Lippincott Company. 1897.

It is almost unavoidable, from the nature of the subjects it deals with, that this volume should be of less general interest than the other three of the series. The sections relating to the examination of the eye naturally suffer from this disadvantage most of all; and recognising this, the authors have in some instances apparently cut down their articles to the smallest possible limits. For example, in the chapter on the methods of determining acuity of vision there is no reference whatever to the important question of estimation of light-sense. The information afforded about mydriatics and myotics is in some respects hardly sufficient. The various theories of the precise method of action of drugs upon the pupil are not given, and it is a matter of regret that the strength of solutions is not stated in percentage. Atropinism—more generally known as atropine irritation—is important enough to deserve a fuller description. Mention is made of arecoline, a myotic of remarkable activity and likely to be of great practical use. The section on the art of ophthalmoscopy is not very satisfactory, and gives the impression that it has been introduced for the sake of theoretical completeness rather than practical utility. Certain essential ophthalmoscopic appearances are very inadequately treated. For example, brief allusion to physiological and glaucomatous cupping of the disc, unless supported by diagrams and illustrations, is utterly useless: the important fact that normal retinal "vessels," as seen with the ophthalmoscope, are in reality hardly more than columns of blood is not brought out at all, and the "pathetic interest" of the albinotic fundus has not induced the author to deal with the various remarkable appearances of the choroid as fully as they deserve. The statement that the whiteness of the optic disc is "due to the myeline sheathing of the fibres, which is discontinued at the papilla border," is not in accordance with microscopic investigation, clinical appearance, or even ordinary text-book information. Were the myeloid sheath continued to the papilla border the lamina cribrosa would be hidden and the characteristic appearance of the healthy disc entirely altered. These and other numerous important omissions will be supplied no doubt in subsequent portions of the work; in which case, the chapter on ophthalmoscopy will be rendered to some extent redundant. The articles on ophthalmometry and prisms appear to contain all matters of essential importance, and all that need be known for the ready understanding and practical application of the "shadow test" is contained in a capital article by Dr. Edward Jackson, but it is a pity that his diagrams of the appearance of the "shadow" are not introduced. About one-quarter of the volume is devoted to Dr. Thomas Pooley's translation of Wilbrand's admirable article on perimetry. It is full of references to cases and the diagrams show the conditions found in almost every ocular or cerebral condition. Curiously enough, there seems to be no reference to the fields so often characteristic of hysteria. It will also be noticed that no modern self-registering perimeter is described, and that the test objects recommended are not those in general use. The chapter on the detection of colour blindness deserves special notice, on account of the growing but still dangerously inadequate attention which is being devoted to the subject. Even a casual perusal of this article will convince anyone that the testing of the colour vision, say of railway servants, should not be left in the hands of an untrained clerk, a practice which is as unfair to the men as it is dangerous to the public.

In a very good article on School Hygiene by Dr. S. D. Risley, dealing with the artificial production of myopia in schools, it is urged that near-sight is not directly hereditary, but that the configuration of the skull is inherited with the astigmatism which it involves: the struggle to improve the sharpness of the image by accommodative effort sets up pathological states which are the foundation of progressive myopia. Referring to a large number of patients repeatedly examined, he says: "My own cases, without exception, passed from the hypermetropic ball over into near-sight through the turnstile of astigmatism. . . . In no instance did these eyes . . . become emmetropic at any stage of their progress." He recommends that as soon as a child can read its vision should be tested and any ametropia corrected. The question of the hygiene of the schoolroom and its furniture, so far as it affects the vision of the children, is carefully considered and illustrated with excellent plans and pictures. The abolition of ordinary desk slates is advocated; and for writing he advises, in preference to the upright style, an oblique position of the paper and consequently the adoption of slanting script, whilst the pupil sits square with the desk.

The volume concludes with a chapter on the micro-organisms of the conjunctival sac. It includes a description of some forty different kinds, most of which apparently have no special significance. The question of Sattler's "trachoma-coccus" is discussed at some length, but the authors conclude that there is not sufficient evidence of any specific bacterium of trachoma.

The volume is fairly well illustrated and seems to be singularly free from misprints, and there are but few variations, such as paralyzant, pathologic, macropia (for macropsia), from the generally accepted word forms. The reason for the arrangement adopted and the narrow limitations of some of the articles is not very evident; but this will no doubt be explained in the two succeeding volumes, to the publication of which we look forward with increased interest.

